# Engineering *Yarrowia lipolytica* to Simultaneously Produce Lipase and Single Cell Protein from Agro-industrial Wastes for Feed

**DOI:** 10.1038/s41598-018-19238-9

**Published:** 2018-01-15

**Authors:** Jinyong Yan, Bingnan Han, Xiaohua Gui, Guilong Wang, Li Xu, Yunjun Yan, Catherine Madzak, Dujie Pan, Yaofeng Wang, Genhan Zha, Liangcheng Jiao

**Affiliations:** 1 Key Lab of Molecular Biophysics of Ministry of Education, College of Life Science and Technology, Huazhong University of Science and Technology, 1037 Luoyu Road, Wuhan, 430074 China; 2 School of Life Science, Zhejiang Sci-Tech University, 928 Second Avenue, Xiasha Higher Education Zone, Hangzhou, 310018 China; 3 GMPA, AgroParisTech, INRA, Université Paris-Saclay, 78850 Thiverval-Grignon, France; 4 MICALIS, INRA, AgroParisTech, Université Paris-Saclay, 78350 Jouy-en-Josas, France; 5 Shanghai Institute of Technology, 100 Haiquan Road, Shanghai, 201418 China

## Abstract

Lipases are scarcely exploited as feed enzymes in hydrolysis of lipids for increasing energy supply and improving nutrient use efficiency. In this work, we performed homologous overexpression, *in vitro* characterization and *in vivo* assessment of a lipase from the yeast *Yarrowia lipolytica* for feed purpose. Simultaneously, a large amount of yeast cell biomass was produced, for use as single cell protein, a potential protein-rich feed resource. Three kinds of low cost agro-industrial wastes were tested as substrates for simultaneous production of lipase and single cell protein (SCP) as feed additives: sugarcane molasses, waste cooking oil and crude glycerol from biodiesel production. Sugarcane molasses appeared as the most effective cheap medium, allowing production of 16420 U/ml of lipase and 151.2 g/L of single cell protein at 10 liter fermentation scale. *In vitro* characterization by mimicking a gastro-intestinal environment and determination of essential amino acids of the SCP, and *in vivo* oral feeding test on fish all revealed that lipase, SCP and their combination were excellent feed additives. Such simultaneous production of this lipase and SCP could address two main concerns of feed industry, poor utilization of lipid and shortage of protein resource at the same time.

## Introduction

Lipases are well known as versatile biocatalysts for various industrial fields including oleochemical, textile, detergent, biodegradable polymers, food additive, biodiesel and so on^1–3^. However, the use of lipases as inducer of oil and fat digestion in feed industry has been relatively ignored.

Feed enzymes are currently focused on phytases, proteases, amylases, and carbohydrases, responsible for releasing phosphorus from phytate, improving protein digestibility, facilitating starch digestibility and decomposing grain-based fibers, respectively^4^. In contrast, lipases are seldom used as feed enzymes. Young animals with immature digestive capability for full absorption of lipids would however particularly benefit from feed lipases. Therefore, there is an increasing trend in feed industry to develop lipases as feed additives. However, most microbial lipases are alkaline properties^5^, thus are generally instable, or even inactivated, in the digestive environment, due to acidity, physiological concentrations of bile salts, and digestive proteases (pepsin, trypsin and chymotrypsin) in intestine and stomach. There remains a need for developing lipase enzymes with resistance to the acidic and digestive environment of intestine and stomach, for feed purpose.

In addition to the low digestion extent of lipids, the insufficient protein-rich feed resources also limits the development of feed industry^3^. Because food related fodder proteins from, for example, grains, beans, meats, fishes, eggs or caseins, compete with human consumption, an alternative source of proteins is required. Thus, single cell protein (SCP), obtained by rapid and efficient growth of protein-rich microorganisms on cheap substrates, is expected to be used as a substitute in animal diets^3,4^. *Yarrowia lipolytica* yeast presents the potential of an excellent fodder yeast^6^.

Moreover, *Y*. *lipolytica* has been proven to be an attractive expression system for homologous and heterologous protein production due to the availability of genetic tools, characteristic, ability to utilize low cost hydrophobic substrates, excellent secretory capability^7^. Its “generally recognized as safe” (GRAS) status makes it especially a good candidate host for producing proteins used in pharmaceutical applications, or in food and feed^6^. Considerable efforts have been made for overexpression of lipase alone, but little attention has been paid to processes for simultaneous production of lipase and other products^8–10^.

**Figure 1:**
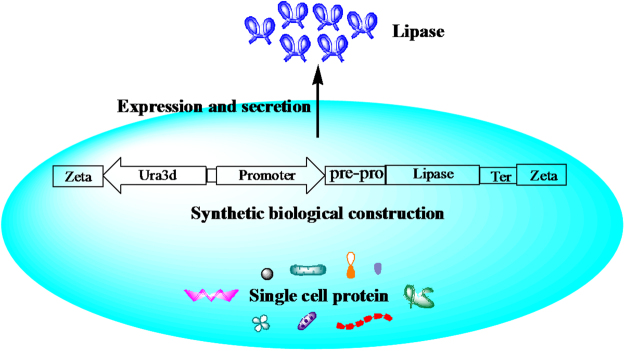
Scheme of lipase enzyme and single cell protein produced by our engineered *Y*. *lipolytica* yeast.

Production of value-added products using waste substrates is a promising strategy from an industrial point of view. In this study, we used *Y*. *lipolytica* as a host for homologous overexpression of an acidic and digestive resistant lipase in different cost-effective agro-industrial waste based media. Through *in vitro* assessment by mimicking a gastro-intestinal environment and characterization of SCP, our purpose is the potential use of this overproduced lipase as a fodder additive, while simultaneously utilizing the host yeast cell biomass as SCP for feed industry (Fig. 1). Such comprehensive utilization of enzymes and yeast biomass based on cheap feedstock for feed could be highly attractive from an industrial perspective. To the best of our knowledge, it is the first time that lipase and SCP were simultaneously produced from cost-effective agro-industrial waste media for feed purpose, especially *in vivo* fish oral feeding assessment.

## Methods

### Strains, plasmids, reagents and media

Wild *Y*. *lipolytica* yeast strain YLY was *i*solated and identified from marine oil-contaminated sludge. *Escherichia coli* DH5α were reserved in our laboratory. Plasmids pINA1317, pINA1297 and pINA1296 have been constructed at INRA (National Institute for Agronomic Research, France) and were described previously^11^. Oligo primers were synthesized by Sangon Biotech (Shanghai, China). All used genetic manipulation reagents and kits were products from Thermo Scientific. Various authentic standards were purchased from Sigma. Pepsin, trypsin, chymotrypsin and sodium deoxycholate monohydrate were purchased from Sigma. The sugarcane molasses with 48.9% sugar content was kindly donated by local sugar manufacturer at Nanning, Guangxi province, China. Waste cooking oil was kindly donated by local restaurant. Crude glycerol was prepared by lipase-catalyzed biodiesel production at our laboratory, and was composed of 84.8% glycerol, 9.8% water, 4.2% glycerides, 0.8% salt and 0.4% protein. Yeast extract and peptone with quite low price were industrial grade. Other routine reagents were commercially available products of analytical grade.

Modified YPD medium was composed of yeast extract (10 g/L), peptone (10 g/L) and glucose (50 g/L). YPS medium consisted of yeast extract (10 g/L), peptone (10 g/L) and sucrose (50 g/L). YPG medium consisted of yeast extract (10 g/L), peptone (10 g/L) and glycerol (50 g/L). YPO medium consisted of yeast extract (10 g/L), peptone (10 g/L) and olive oil (20 g/L). YNBD selective medium was made up of yeast nitrogen base (without amino acids, 6.7 g/L) and glucose (10 g/L). Agar (20 g/L) was added for preparing solid plates. Molasses based broth YPM contained molasses (20 g/L), yeast extract (10 g/L), peptone (10 g/L) and citrate buffer (20 mM, pH 6.0). Crude glycerol based broth YPCG contained crude glycerol (20 g/L), yeast extract (10 g/L), peptone (10 g/L) and citrate buffer (20 mM, pH 6.0). Waste cooking oil based broth YPW contained waste cooking oil (20 g/L), yeast extract (10 g/L), peptone (10 g/L) and citrate buffer (20 mM, pH 6.0). YNBS medium comprised yeast nitrogen base (without amino acids, 6.7 g/L), sucrose (10 g/L) and agar (20 g/L). The 5-fluoro-orotic acid (5-FOA) medium consisted of yeast nitrogen base (without amino acids, 3.5 g/L), 5-FOA (0.5 g/L), uracil (25 mg/L), glucose (10 g/L) and agar (20 g/L).

### Auxotrophic *Y. lipolytica* host strain construction

**Figure 2:**
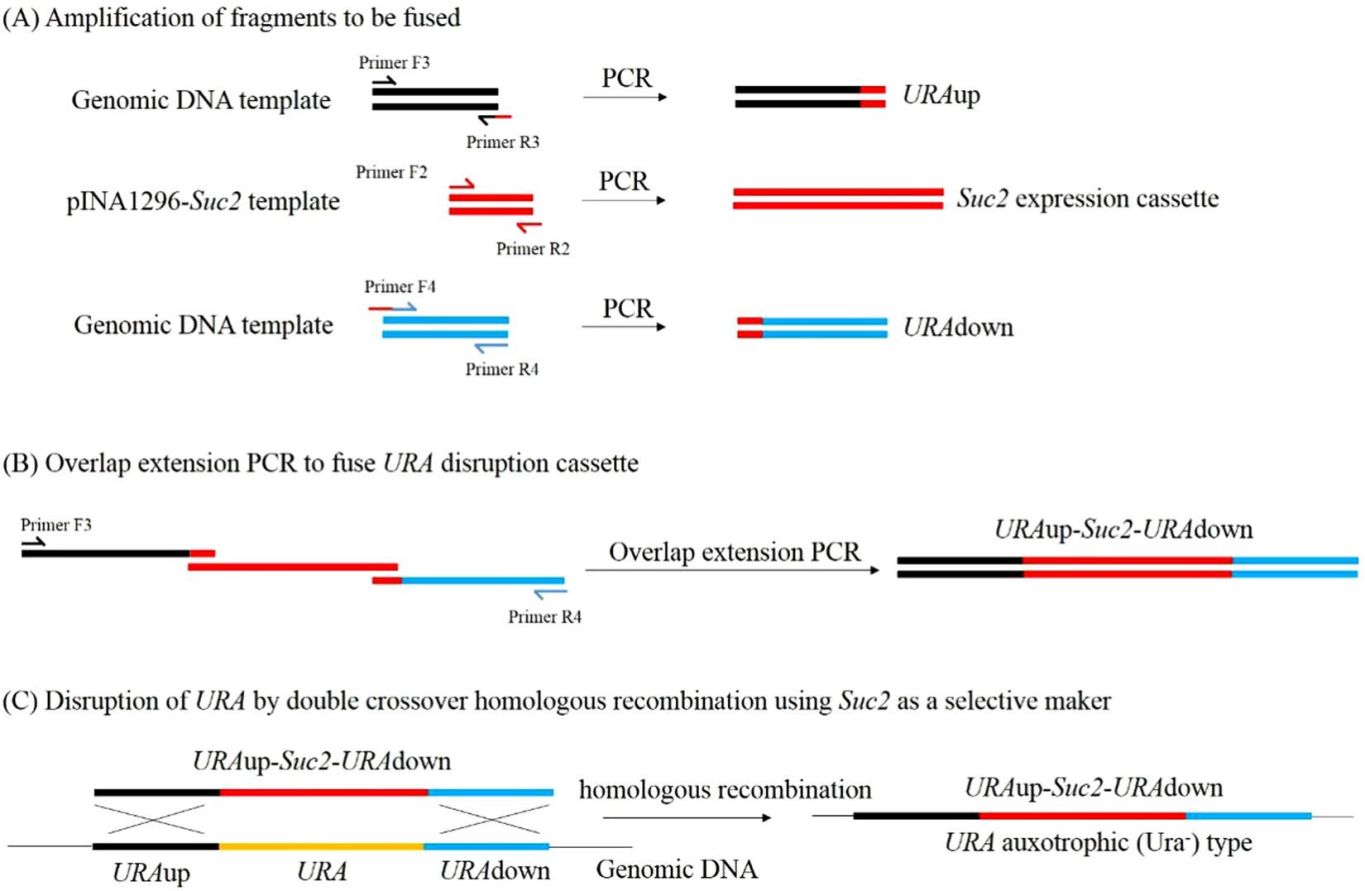
Construction of the auxotrophic strain. (**A**) Amplification of the three fragments to be fused: *URA3* upstream region, *SUC2* expression cassette and *URA3* downstream region, (**B**) Overlap extension PCR fusing *URA*up, *SUC2* expression cassette and *URA* down to form the *URA* disruption cassette, (**C**) Disruption of *URA3* by double crossing over (homologous recombination) using *SUC2* as a selective marker.

Mature invertase gene *SUC2* fragment was amplified from genomic DNA of *S*.*cerevisiae* using primers F1 and R1, and was inserted into *Sfi*I-*Hin*dIII sites of pINA1296 to give rise to a recombinant plasmid pINA1296-*suc2*. *SUC2* expression cassette comprised of hp4d promoter, *XPR2*pre sequence, *SUC2* gene and *XPR2* terminator was amplified using plasmid pINA1296-*suc2* as the template and F2 and R2 as primers. The *URA3* upstream (*ura*-up) and downstream (*ura*-down) homologous fragment regions were amplified from genomic DNA of *Y*. *lipolytica* YLY using F3 and R3, F4 and R4 as primers, respectively (Fig. 2A). The primers R3 and F2, as well as R2 and F4 shared 20 bp overlap oligonucleotides for overlap extension PCR (Table 1). The three PCR fragments (*ura*-up, *SUC2* expression cassette, *ura*-down) were fused to form *URA3* gene disruption cassette (up-Suc2- down) using primers F3 and R4 by overlap extension PCR technique (Fig. 2B). The disruption cassette (up-Suc2-down) was transformed into competent cells of *Y*. *lipolytica* YLY by previously described method^12^. The positive transformant selected on both YNBS and 5-FOA media was called auxotrophic *Y*. *lipolytica* host strain and used for lipase overexpression.

**Table 1:** Primers used in this study.

Primer	Sequence
F1	TCACGGCCGTTCTGGCCGGTTTTGCAGCCAAAATATCTGCA
R1	TAGAAGCTT CTATTTTACTTCCCTTACTTGGAACTT
F2	AGTCTCCTCTTCACCACCAAAGCTCTCCCTTATGCGACTCC
R2	TATCCATAGTCTAACCTCTAGGACACGGGCATCTCACTTG
F3	ACTGGCCAAACTGATCTCAAGACTTTATTGAAATCAGCAA
R3	GGAGTCGCATAAGGGAGAGCTTTGGTGGTGAAGAGGAGACT
F4	CAAGTGAGATGCCCGTGTCCTAGAGGTTAGACTATGGATA
R4	GAAGGGGCCTATAATGCCTCTTTTTTAAGTTCCTGGAGC
F5	TACGGCCGTTCTGGCCATGAAGCTTTCCACCATCCT
F6	TACGGCCGTTCTGGCCCTGGCTGCCGCCCTCCCTTCCCCC
F7	TACGGCCGTTCTGGCCATCACTCCTTCTGAGGCCGCAGTT
F8	TACGGCCGTTCTGGCCGTGTACACCTCTACC
R5	CGGGGTACCTTAGATACCACAGACACCCTCGGT

*URA3* gene encoding orotidine-5’-phosphate decarboxylase, a key enzyme for uracil biosynthesis, was disrupted by homologous recombination in YLY strain. Coding sequence for *URA3* gene was replaced by *SUC2* expression cassette via homologous recombination based on double crossover events (Fig. 2C). The successful disruption of *URA3* was selected by the capacity of disrupted cells to grow on medium containing 5-fluoro-orotic acid (5-FOA), a pyrimidine analog. Wild type *Y*. *lipolytica* cells harboring functional *URA3* are unable to grow on 5-FOA containing medium due to formation of a toxic product catalyzed by functional *URA3*^16^. Moreover, the positive clones selected from 5-FOA containing medium were further screened on YNBS medium, with sucrose as sole carbon source.

### Homologous overexpression of lipase

Several versions of the lipase gene were assayed: the complete gene (*pre-dp-pro-mlip*), truncated genes with removal of either the pre-sequence (*dp-pro-mlip*) or of the pre-dp-sequence (*pro-mlip*), and the mature lipase gene (*mlip*) alone. These various *lip* related fragments were cloned from genomic DNA of *Y*. *lipolytica* YLY using F5 and R5, F6 and R5, F7 and R5, or F8 and R5 as primer pairs, respectively. The series of *Sfi*I-*Kpn*I double digested *lip* related fragments were inserted into the corresponding sites of plasmids pINA1312 and pINA1317, resulting in monocopy integrative plasmids pINA1312-*pre-dp-pro-mlip*, pINA1317-*dp*-*pro- mlip*, pINA1317-*pro-mlip* and pINA1317-*mlip*, respectively. To assess the effect of gene copy number on lipase production, a multicopy integrative plasmid, pINA1297-*pro-mlip*, was also constructed. After sequence confirmation, the *Not*I-digested recombinant plasmids were used to transform competent cells of the auxotrophic *Y*. *lipolytica* host strain. Monocopy integrated strain (based on pINA1312 and pINA1317) and multicopy integrated strain (based on pINA1297) were selected by growth on minimal medium YNBD. Fermentation of the resulting recombinant *Y*. *lipolytica* strains was performed in various media including modified YPD, YPS and molasses based broth for investigation of extracellular lipase and cell biomass production.

### Fermentation process

A fermentation process for lipase and SCP production by the best engineered *Y*. *lipolytica* strain was performed in 10 liter bioreactor. The bioreactor loaded with 4 liter YPM medium was inoculated by 400 ml pre-culture of the *Y*. *lipolytica* strain. The fermentation was carried out at 28 °C with a stirring speed of 300–600 rpm and an air flow of 1–4 liter/min to ensure a dissolved oxygen of 30% air saturation. pH was maintained at 6.0 ± 0.2 by addition of ammonium hydroxide. Antifoam (silicone emulsion) was initially added to the medium before sterilization to avoid excessive foaming.

### Preparation of lipase enzyme formulation with addition of protective additives

The liquid cell free supernatant containing lipase from fermentation broth was supplemented with a variety of 1% additives (gum arabic, skimmed milk powder, maltodextrin, CaCl_2_ and glycerol) and stored at −80 °C for 24 h to form solids. Subsequently, the solids were lyophilized at −50 °C using freeze-dryer until obtaining uniform powders. The protection effects of the additives on lipase were characterized by the residual activities present in the lyophilized powders. The lipase-containing supernatant without any additives was used as the control. The activity of original supernatant without any freeze-drying treatments was set as 100%.

### Lipase activity, cell biomass (single cell protein), crude protein content and amino acid composition assay

The engineered strains were pre-cultured in 5 ml YPD medium under shaking at 250 rpm and 28 °C for 12 h. These seed cultures were used to inoculate 100 ml of various media (including YPD, YPS, YPG, YPCG, YPO, YPM, YPW) contained in 500 ml flask and cultivated at 250 rpm and 28 °C for designate times. The culture supernatant and cell pellets were separated by centrifugation (2000 *g*, 5 min). The culture supernatant was used for detection of lipase activity. The harvested cell pellets were washed two times with sterile water, and then dried to constant weight at 105 °C for determination of cell dry weight. The single cell protein was presented as cell dry weight. Dried cells were disrupted by mortar for pounding, and crude protein content was measured by the Kjeldahl method based on quantitative determination of released nitrogen from the amine groups in the protein polypeptide chains^13^. Amino acid composition was determined on automatic amino acid analyzer following the standard procedure^14^. Lipase activity assay was performed by titration method as previously described^15^. The titrimetric assay was based on using emulsified olive oil as substrate and determination of released free fatty acids by titration against potassium hydroxide. Enzyme protein was determined using protein assay kit according to instruction manual.

### Oral effect of lipase and SCP on growth of Cynoglossus semilaevis gunthers

A kind of marine fish, Cynoglossus semilaevis gunthers with 46–56 g body weights were involved in oral effect experiments during 32-day feeding. These fish were treated with various doses of lipase, SCP and their combination that were mixed with the diet. They ranged in 2–12 U lipase per gram diet and 0.5%-3% of SCP based on diet weight, respectively. These fish were divided into different groups according to the corresponding feed diets. Each group set four repetitive tanks, and each tank had 1700 fish. Their mean values presented as experimental results. These different diets containing various doses of lipase, SCP and their combinations were compared to a fish meal based control diet (fish meal 55%, shrimp meal 20%, fish oil 5%, crude fat 13.3%, phospholipid 1.7%, starch 1%, betaine 0.3%, pigment 0.1%, vitamin mix 0.8%, mineral mix 0.8%). Oral effect of lipase and SCP on fish weights and growth rates were characterized by relative weight gain (RWG) and specific growth rate (SGR), respectively. RWG = (W_t_ − W_0_)/W_0_ × 100%, SGR = (lnW_t_ − lnW_0_)/t × 100%, where W_0_ and W_t_ are body weights at start and end of the experiments, and t is feeding time.

All experimental protocols were approved by Wenhai fish farm (Weifang, China). All the *in vivo* feeding experiments were performed in Wenhai fish farm (Weifang, China) according to approved guidelines and regulations of evaluation of fish feed additives.

## Results

### Expression of lipase

The wild type *Y*. *lipolytica* strain YLY could secrete extracellular lipase (7 U/ml) in modified YPD medium containing 1% olive oil (v/v). According to previous reports, lipase 2 (Lip2) was found to be the main contributor to extracellular lipase activity in *Y*. *lipolytica* strains^17^. Complete cDNA sequence of the gene *lip2* encodes a lipase enzyme composed of a 13 amino acid signal peptide (pre-), four dipeptides (X-Ala or X-Pro) that are cutting sites of a diamino peptidase (dp-), a 12 amino acid pro-region containing a Xpr6 endoprotease site Lys-Arg (pro-), and a 301 amino acid mature lipase (mlip). Therefore, based on the previously reported *lip2* sequence deposited under NCBI accession number AJ012632, we designed and used F5 and R5 primers, that were able to amplify a lipase gene *lip* of 1005 bp (accession number KY197472) from YLY strain genomic DNA. The amino acid sequence of *lip* showed very high similarity to Lip2, with 89% identity. A series of *lip* gene related constructs with either complete gene or truncated versions with removal of pre sequence, dp sequence, and/or pro sequence were generated and inserted into monocopy integrative vectors (pINA1312 and pINA1317) or into a multicopy integrative vector (pINA1297). These plasmids are “auto-cloning” expression vectors, which means that a restriction digestion (using *Not* I enzyme) is performed to separate the bacterial moiety from the “yeast cassette” that will be used for transformation of the *Y*. *lipolytica* strain^18^. This “yeast cassette”, composed of an *ura3d1* or *ura3d4* (defective) selection marker allele and one of the different *lip* expression cassettes, is able to confer to the auxotrophic *Y*. *lipolytica* host strain Ura^+^ phenotype, allowing the transformants to grow on selective medium YNBD. The term “auto-cloning” reflects the fact that the selected transformants have integrated only the “yeast cassette” and do not bear any DNA from bacterial origin (and especially no antibiotic resistance gene).

**Figure 3:**
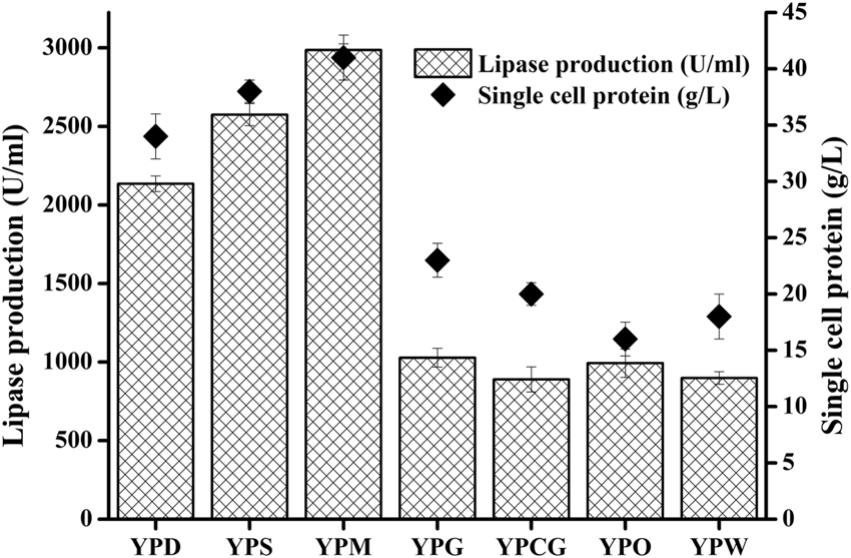
Evaluation of different culture media, based on pure carbon sources or on low cost agro-industrial waste substrates (sugarcane molasses, waste cooking oil and crude glycerol) for simultaneous production of lipase and single cell protein. Lipase activity and SCP yield are the mean ± SD of three independent assays.

A secretion signal peptide (*xpr2pre*) derived from alkaline extracellular protease encoded by *xpr2* gene was frequently used for heterologous expression of protein in *Y*. *lipolytica*^19^. Therefore, both *xpr2pre* and Lip’s own secretion signal (*lippre*) were compared for extracellular lipase production. As presented in Table 2, comparison of engineered strains YLY1 and YLY2 indicated that *xpr2pre* was more effective than *lippre* in directing lipase into extracellular medium. Therefore, *xpr2pre* was used for secretion of lipase in subsequent constructs. Comparison of engineered strains containing different truncated versions of *lip* gene (YLY2, YLY3, YLY4) demonstrated that deleting the dipeptides while keeping the Lip2 pro-region was the most favorable combination for extracellular lipase production (Table 2). To investigate the effect of gene copy number, lipase expression from monocopy integrative vector pINA1317 and multicopy integrative vector pINA1297 were compared. The only difference between these two vectors is that pINA1297 carries a defective *ura3d4* selection marker allele, expected to be unable to confer Ura^+^ phenotype when present in a single copy^11^. Our results showed that pINA1297 based higher gene copy number integration led to an extracellular lipase activity of 2175 U/ml (Table 2), which represents a 1.4-fold increase compared to the pINA1317 based monocopy counterpart, and a 311-fold increase compared to wild type *Y*. *lipolytica* strain YLY. The highest lipase-producing strain YLY5 was selected for further studying of lipase production in modified YPD, YPS, YPO and YPG media. Testing this array of media with different carbon sources revealed a wide spectrum of carbon utilization in this yeast strain. The recombinant YLY5 strain could secrete extracellular lipase with 2175 U/ml activity in modified YPD medium, 2615 U/ml activity in YPS medium, 1028 U/ml activity in YPG medium and 993 U/ml activity in YPO medium, after 96 h of cultivation (Fig. 3). The SCP yield varied consistently with lipase production. The high cell biomass attained could contribute to overall lipase production. Considering its economic viability and the high production of lipase obtained, the sucrose based YPS medium was selected for further study.

**Table 2:** Comparison of different constructs.

Engineered strain	Plasmid backbone	Expression cassette elements	Selection marker	Activity (U/ml)^a^
YLY1	pINA1312	hp4d/*lippre-dp-pro-mlip/xpr2*t	*ura3d1*	226 ± 4
YLY2	pINA1317	hp4d/*xpr2pre-dp-pro-mlip/xpr2*t	*ura3d1*	1150 ± 5
YLY3	pINA1317	hp4d/*xpr2pre-pro-mlip/xpr2*t	*ura3d1*	1550 ± 3
YLY4	pINA1317	hp4d/*xpr2pre-mlip/xpr2*t	*ura3d1*	987 ± 3
YLY5	pINA1297	hp4d/*xpr2pre-pro-mlip/xpr2*t	*ura3d4*	2175 ± 2

^a^The engineered strains were cultivated in modified YPD medium. Lipase activity is the mean ± SD of three independent assays.

### Characterization of lipase in terms of feed enzyme features

**Figure 4:**
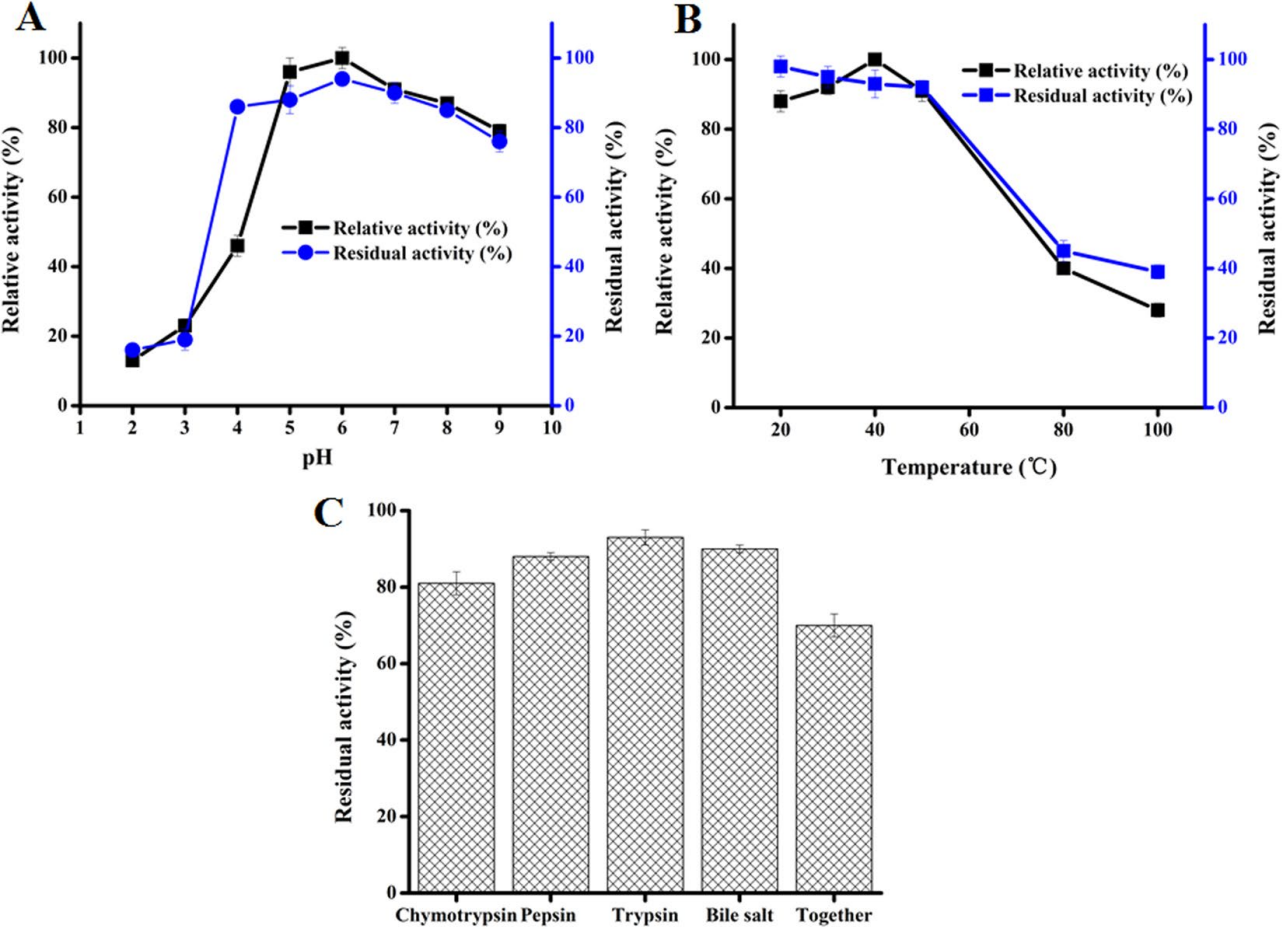
Characterization of lipase in term of feed enzyme features. (**A**) Effect of pH on lipase activity, (**B**) Effect of temperature on lipase activity, (**C**) *In vitro* evaluation of the stability of lipase in condition mimicking gastro-intestinal section including acid pH, in the presence of bile salt or/and digestive proteases. Lipase activity is the mean ± SD of three independent assays.

Stability of lipase was evaluated *in vitro* in conditions mimicking those of gastro-intestinal section, namely acid pH or presence of bile salt or digestive proteases. As shown in Fig. 4A, lipase displayed its highest activity at pH 6, and maintained more than 86% of residual activity at pH 4–8 and 16% of residual activity at pH 2–3 after incubation at various pH for 30 min. This demonstrates that the overproduced lipase possesses excellent acid stability, an important characteristic for a feed enzyme. Optimal temperature of this enzyme was found to be 40 °C, and it retained more than 92% of residual activity after incubation at 50 °C for 30 min. More interestingly, this lipase exhibited 39% of residual activity after treatment at 100 °C for 30 min (Fig. 4B), which demonstrated its superior thermostability, a highly desirable feature during downstream processing, such as spray-drying, for industrial production.

As shown in Fig. 4C, the sensitivity of feed lipase to digestive proteases was evaluated. The lipase exhibited 69%, 88% and 93% of residual activities after incubation with chymotrypsin (5 mg/ml), pepsin (3.2 mg/ml) and trypsin (5 mg/ml), respectively. Also, the lipase retained more than 90% of residual activity in the presence of bile salt (2 mM).

A mixture containing 5 mg/ml chymotrypsin, 3.2 mg/ml pepsin, 5 mg/ml trypsin and 2 mM bile salt in buffer (pH 4) was used for mimicking gastro-intestinal environment. Cell culture supernatant (100 μl) containing 337.5 U lipase and 5% (w/v) soybean oil were added to the above digestion mimicking mixture, in order to investigate oil utilization, by measuring residual activity and hydrolysis extent. Significantly, 72% of residual activity was detected in the mixture (Fig. 4C), and 81% of the oil was hydrolyzed to free fatty acids after 10 min at 37 °C.

### Effect of additives on freeze-dried lipase formulation

**Figure 5:**
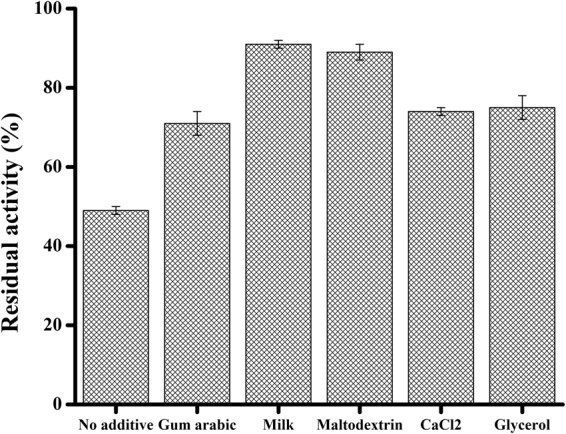
Effect of several additives on freeze-dried lipase formulation. Lipase activity is the mean ± SD of three independent assays.

Freeze-drying of supernatant containing lipase to obtain a stable formulation is a key step in the downstream process of feed lipase preparation. However, enzymes tend to change their physical and chemical properties and lose some activity during the freezing and drying process. In this study, various additives including gum arabic, skimmed milk powder, maltodextrin, CaCl_2_ and glycerol were investigated for their stabilizing effects on lipase powder. As presented in Fig. 5, maltodextrin and milk powder (1%) were found more effective in stabilizing lipase powder during freeze-drying, as shown by higher residual activity. This could be explained by the fact that casein from milk powder could promote interaction of the enzyme with hydrophobic substrate at the lipid-water interface by forming hydrogen bonds with some lipase residues. Also, oligosaccharides present in maltodextrin could maintain the conformation of enzyme during the freeze-drying process.

### Simultaneous production of lipase and SCP from sugarcane molasses

In the present study, the low cost substrate molasses was utilized by genetically engineered strain YLY5 for producing high value added lipase and SCP. In combination with identical amounts of yeast extract (10 g/L) and peptone (10 g/L), various carbon sources, including glucose, sucrose and sugarcane molasses (present in respectively YPD, YPS and YPM media), were evaluated in terms of lipase and SCP yields. As described in Fig. 3, molasses based broth was found to be superior to other broths such as YPD and YPS for both lipase (3075 U/ml) and SCP production (39 g/L). Results were also better in YPS than in YPD, leading to the conclusion that the invertase harboring yeast utilized sucrose more effectively than glucose.

### Simultaneous production of lipase and SCP from waste cooking oil

In this study, WCO was employed as a carbon source for lipase and SCP production by engineered *Y*. *lipolytica*. As shown in Fig. 3, the lipase production in WCO broth (980 U/ml) was comparable to that in much more expensive olive oil broth YPO (993 U/ml). The SCP yield based on WCO broth (17.9 g/L) was slightly higher than that in olive oil broth (15.6 g/L). However, this difference in growth was not reflected in lipase production, which was slightly higher in YPO. A comprehensive consideration of both lipase-SCP production and feedstock cost led to the conclusion that WCO broth would be more advantageous for feed yeast production.

### Simultaneous production of lipase and SCP based on raw glycerol derived from biodiesel production

We compared different carbon sources, including pure glycerol, raw glycerol, glucose and sucrose for lipase and SCP production. As shown in Fig. 3, pure glycerol gave higher SCP yield (22.3 g/L) and lipase production (1028 U/ml) than crude glycerol (SCP 19.7 g/L, lipase 969 U/ml). The yields show that raw glycerol can be used for feed yeast production. However, the yields of SCP and lipase were lower than when using sugar-based carbon sources.

### Simultaneous production of lipase and SCP at 10 liter fermentation scale

**Figure 6:**
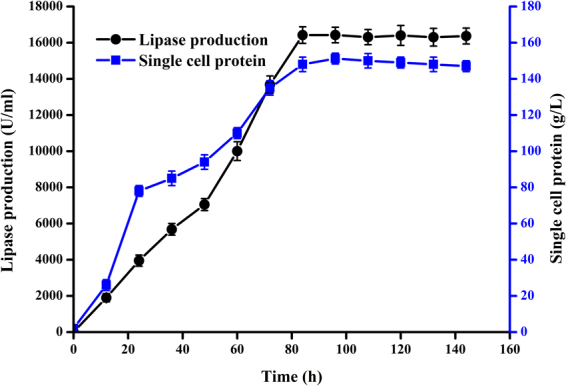
Time course of lipase and SCP production at 10 liter fermentation scale.

Lipase activity and SCP were monitored at regular time points during 10 liter fermentation process. As described in Fig. 6, the SCP increased during first 95 h to achieve the highest yield of 151.2 g/L, and maximal enzyme production with 16420 U/ml (4.13 g/L) was observed at 89 h. During the fermentation process, the lipase production increased with the increase of cell biomass. The lipase production and SCP yield far exceeded previous reports^18,21,22^.

### Characterization of SCP produced by *Y*. *lipolytica* yeast

Protein content, amino acid composition and amino acid concentration are all important factors affecting the quality of fodder additives. The crude protein content of *Y*. *lipolytica* ranged from 45% to 54% depending on media composition and culture conditions. Analysis of amino acid composition showed that the SCP extracted from *Y*. *lipolytica* biomass contained essential amino acids required for animal feed, as issued by The Food and Agriculture Organization of the United Nations (FAO)^4,23^. More importantly, with the exception of cystine, the concentrations of lysine, threonine, cysteine, valine, methionine, isoleucine, leucine, tryptophan and phenylalanine were comparable to the standards recommended by the FAO (Table 3), which demonstrates that *Y*. *lipolytica* biomass constitutes a suitable fodder resource for feed industry.

**Table 3:** Comparison of essential amino acid composition between *Y*. *lipolytica* yeast and FAO standard.

Essential amino acid	Concentration (% of total protein)	
	*Y*. *lipolytica* yeast ^a^	FAO standard ^b^
Lysine	4.13 ± 0.03	4.2
Threonine	3.12 ± 0.04	2.8
Cystine	1.34 ± 0.02	2.0
Valine	4.11 ± 0.05	4.2
Methionine	2.32 ± 0.02	2.2
Isoleucine	4.41 ± 0.06	4.2
Leucine	5.13 ± 0.07	4.8
Tryptophan	1.32 ± 0.04	1.4
Phenylalanine	2.63 ± 0.05	2.8

^a^Essential amino acids concentrations are means ± SD of three independent measurements.

^b^From Kurbanoglu and Algur (2002).

### Oral effect of lipase and SCP on growth of Cynoglossus semilaevis gunthers

**Figure 7:**
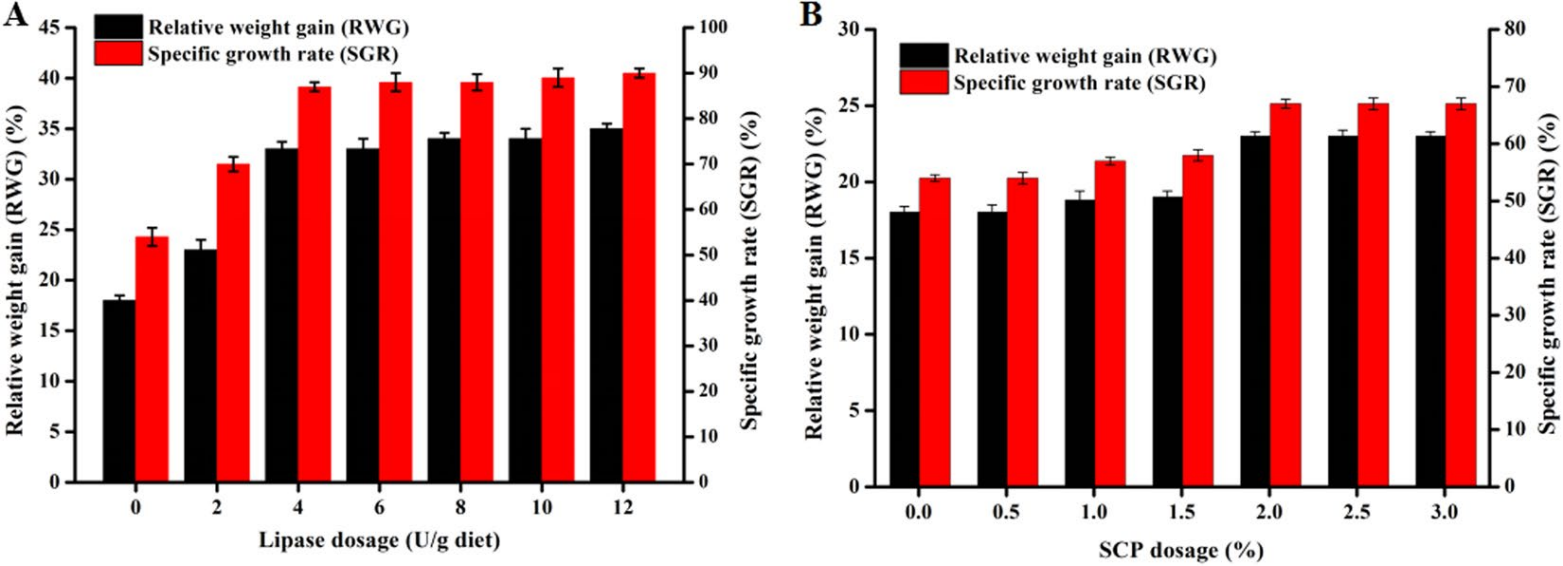
Oral effect of lipase (**A**) and SCP (**B**) on growth of Cynoglossus semilaevis gunthers. The data of RWG and SGR were obtained after 32 d growth with diets containing various dosages of lipase and SCP, and presented as the mean ± SD of three independent assays.

After 32 days of feeding, average weights in fish fed the 4 U/g and 8 U/g lipase-containing diets were significantly higher compared to fish fed the control diet, reflected as higher RWG. In the cases of 12 U/g lipase-containing diets, the weight enhancement was not further increased. The weight enhancement was not significant for 2 U/g lipase-containing diet group. Similar lipase contribution to SGR was also observed (Fig. 7A). The different effect of lipase on RWG and SGR could be explained by that 2 U/g lipase is insufficient for mobilization of lipid present in diet, and 12 U/g lipase is excessive, and 4–8 U/g lipase is adequate for lipid digestion. We also assessed other *Thermomyces lanuginosus* lipase and *Candida antarctica* lipase B as additives for feeding, however no improvement in RWG and SGR was observed (data not shown).

Diets with different addition level of SCP were also evaluated. Appropriate amount of SCP added to diets promoted fish growth, as well as SGR (Fig. 7B). More than 2% SCP addition displayed no further positive effect. Less than 2% SCP addition is insufficient, whereas more than 2% SCP is sufficient for growth promotion due to the potential various functional components (crude proteins, nucleic acids, lipids, carbohydrates, vitamins, mineral elements etc.) present in SCP. We found the combination of 4–8 U/g lipase and 2% SCP further enhanced the SGR and RWG. In addition to the very positive of lipase and SCP effect on fish growth, interestingly, the low toxic effect was reflected by low number of fish deaths. The results showed that simultaneous products of lipase and SCP generated in one single fermentation process were excellent additives for fish feeding.

## Discussion

The wild type *Y*. *lipolytica* could not metabolize sucrose. In disrupted cells, heterologous expression of *SUC2* conferred a Suc^+^ phenotype, which demonstrated that *SUC2* gene could be used as a selective marker for gene disruption^24,25^. From an industrial perspective, sucrose-utilizing microorganisms show significant advantage in reducing the price of carbon feedstock, especially by using cheap sugarcane molasses waste.

Conversion of low cost substrates to value added products by *Y*. *lipolytica* could generate very attractive bioprocesses. Molasses is a by-product during sugar making from sugarcane and beet^26^. Waste cooking oil (WCO) is a kind of low quality grade and much less expensive oil recovered from food industry^27^. Biodiesel production through transesterification of various oils with methanol produces crude glycerol as an abundant by-product^28–30^. In the present study, a genetically engineered *Y*. *lipolytica* strain was employed for studying simultaneous production of lipase and SCP in low cost media based on molasses, crude glycerol and waste cooking oil. The slightly higher SCP yield based on WCO broth than olive oil broth could be explained by the fact that some amounts of free fatty acids present in WCO supported yeast growth more effectively, due to easier utilization efficiency, than olive oil composed mainly of triacylglycerol^20,27^. We also infer that small amount components present in raw glycerol are not particularly detrimental and that some (glycerides, salts and proteins) are probably used as combined carbon and nitrogen sources for growth and also possibly as inducers for lipase expression^28,30^. Furthermore, simultaneous production of lipase and SCP using cost-effective YPM broth at 10 liter fermentation scale with much higher yields than previous reports confers economic vaibility. The possible reasons exist in molasses used in the present study, and pure glucose, glycerol served as carbon sources in previous studies. Multiple components such as glucose, fructose, small amount of mineral substances and bioactive compounds present in sugarcane molasses could benefit to yeast growth and enzyme production.

Most microbial lipases have alkaline properties and are thus not being explored for feed purpose^5^. *T*. *lanuginosus* lipase and *C*. *antarctica* lipase B just showed lower than 10% of residual activities in the conditions mimicking gastro-intestinal section, namely acid pH or presence of bile salt or digestive proteases (data not shown). The excellent tolerance of our lipase in the presence of digestive components, in conditions mimicking a gastro-intestinal environment, showed the potential of this lipase for use as a feed enzyme.

*In vivo* fish oral feeding assessment showed both positive effects of lipase and SCP on growth of Cynoglossus semilaevis gunthers. The lipase facilitated the hydrolysis of lipid present in fish diet and improved utilization of lipid to support fish growth through energy supply and storage, as well as for providing necessary fatty acids. SCP mainly supplemented essential nutrition components in animal growth through essential amino acids supply. To improve digestion and bioavailability of these nutrient ingredients, this lipase and SCP are accepted as feed additives.

## Conclusions

Taken together, a *Y*. *lipolytica* strain was engineered for overproduction of lipase, and simultaneous production of lipase and SCP on cost-effective media. The resulting lipase and SCP were developed for fodder purpose through *in vitro* characterization of its feed characteristics and *in vivo* fish oral feeding assessment. Through an auxotrophic host strain construction, the optimization of signal peptide and increased gene dosage, the best engineered strain produced simultaneously 16420 U/ml of lipase and 151.2 g/L of SCP, using molasses broth as medium at 10 liter bioreactor. The compatibility of the secreted lipase with mimicked gastro-intestinal environment, the concentration in essential amino acids of the SCP, and the very positive oral effect all demonstrated this engineered yeast as an excellent feed resource.
